# Sjögren's Syndrome Overlap With Lupus Nephritis: A Case Report and Literature Review of a Rare Entity

**DOI:** 10.7759/cureus.38666

**Published:** 2023-05-07

**Authors:** Sujith K Palleti, Maria M Picken, Anuradha Wadhwa

**Affiliations:** 1 Department of Internal Medicine/Nephrology, Edward Hines Jr. Veterans Administration Hospital, Hines, USA; 2 Department of Internal Medicine/Nephrology, Loyola University Medical Center, Maywood, USA; 3 Department of Pathology, Loyola University Medical Center, Maywood, USA

**Keywords:** sjögren's syndrome/sle overlap, revised acr/eular criteria, overlap syndromes, sle and lupus nephritis, immune complex mpgn

## Abstract

Overlap syndrome is a connective tissue disorder that satisfies the diagnostic criteria for at least two well-known autoimmune diseases. In this report, we describe a rare case of lupus overlap in an elderly woman with primary Sjögren's syndrome who presented with features of nephritic-nephrotic syndrome and renal biopsy results typical of lupus nephritis combined with multiple positive autoantibodies. The kidney biopsy results were assigned the highest weightage based on the revised 2019 systemic lupus erythematosus (SLE) classification criteria developed by the European League Against Rheumatism (EULAR) and the American College of Rheumatology (ACR). The patient's condition significantly improved after appropriate immunosuppressive therapy was initiated. With the revised ACR/EULAR-2019 criteria, we anticipate that more SLE patients with typical lupus nephritis biopsy findings will be diagnosed accurately.

## Introduction

The term "connective tissue disease" refers to a group of disorders affecting the protein-rich tissues that support the body's organs and other structures, such as fat, bone, and cartilage. Among connective tissue disorders, overlap syndrome refers to a disease entity that meets the diagnostic requirements for at least two well-known autoimmune diseases [1]. Overlap syndrome, which comprises autoimmune disorders such as polymyositis, dermatomyositis, rheumatoid arthritis, and Sjögren's syndrome, is most commonly found in conjunction with systemic lupus erythematosus (SLE)/systemic scleroderma (SSc) [2]. Since patients with overlap syndrome share many common clinical and immunologic characteristics, its diagnosis is frequently challenging [3,4]. One syndrome may start several years before another and not manifest new symptoms until much later [4]. Therefore, it is crucial to recognize such syndromes as soon as possible to provide appropriate care. SLE is a chronic autoimmune illness with no known cause that can affect nearly every organ. The updated SLE criteria, developed by the American College of Rheumatology (ACR) and the European League Against Rheumatism (EULAR), were introduced in 2019 and have been externally verified with a sensitivity and specificity of 96% and 93%, respectively [5].

## Case presentation

A 75-year-old Caucasian female with a past medical history of chronic kidney disease stage 3, hypertension, and primary Sjögren's syndrome diagnosed in 2009 with regular rheumatology follow-up presented to the renal clinic as part of the follow-up with complaints of worsening renal function, proteinuria, microscopic hematuria, and recent spontaneous deep vein thrombosis (DVT) of the left leg. Before the presentation, the following antibodies had been detected as positive: Sjögren's syndrome A (SS-A/Ro), Sjögren's syndrome B (SS-B/La), anti-ribonucleoprotein (antiRNP), anti-nuclear antibody (ANA), and anti-Smith (Sm). Additionally, she exhibited both low normal complement C3 and chronically low complement C4. Cryoglobulins, hepatitis B, and hepatitis C virus testing were negative.

The clinical examination was unremarkable, except for a small amount of stable pitting edema in the ankles, which was unchanged. Laboratory tests performed during the visit revealed nephrotic range proteinuria at 6 g/g, hypoalbuminemia of 2.8, worsening renal function with an increase in serum creatinine to 1.81 mg/dl from a baseline of 1-1.2 mg/dl, new-onset hematuria with a large amount of blood in the urinalysis, RBC of 57/hpf, anemia with hemoglobin of 8.7 mg/dl, and lymphopenia. Serological tests revealed positive results for the lupus anticoagulant, positive MPO-ANCA with titers of >1:640, positive ANA with titers of >1:1280, low C4 at 13 mg/dl, and normal C3. DsDNA was negative. The serum free light chain ratio and serum protein electrophoresis (SPEP) were both normal.

**Figure 1:**
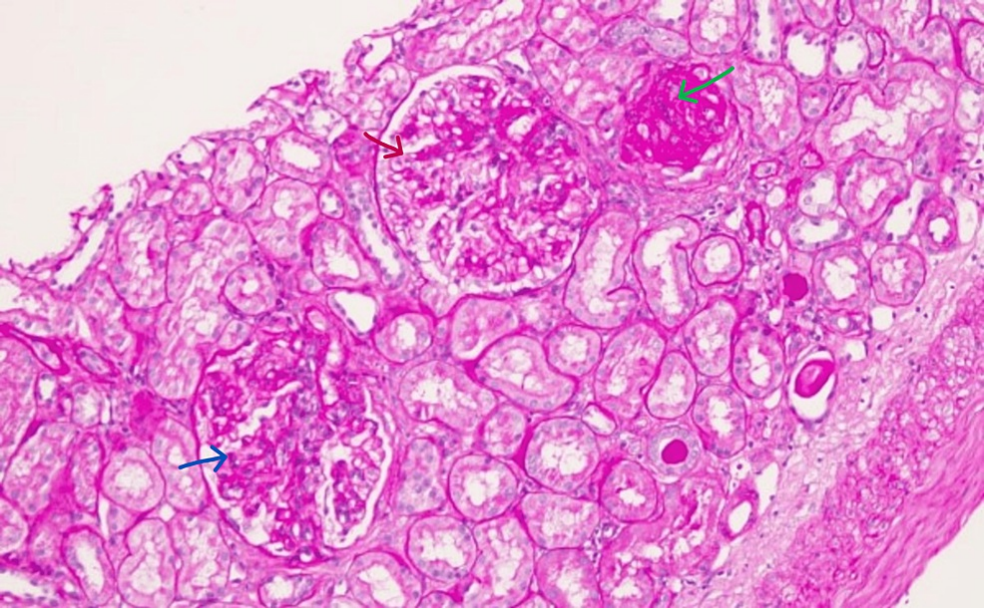
Kidney biopsy Paraffin sections show two hypercellular glomeruli, one of which with lobular tuft (blue arrow) and the other with moderate mesangial expansion (red arrow); one glomerulus is globally sclerosed (green arrow) PAS stain, original magnification 100x

Following a renal biopsy, it was discovered that 25% of glomeruli (14/57) had developed global sclerosis; the majority of the non-sclerosed glomeruli were hypercellular with tufts frequently with lobular accentuation; there was also thickening of the glomerular capillary wall, and mesangial expansion (Figure 1). Up to five glomeruli (9%) showed only mild mesangial expansion. There was one cellular crescent involving 50% of Bowman’s capsule circumference and three fibrocellular crescents involving 60% (x1) of Bowman’s capsule circumference and two circumferential fibrous crescents. There were small foci with tubular atrophy involving <25% of the cortex and mild subintimal fibrosis in the extraglomerular vessels.

**Figure 2:**
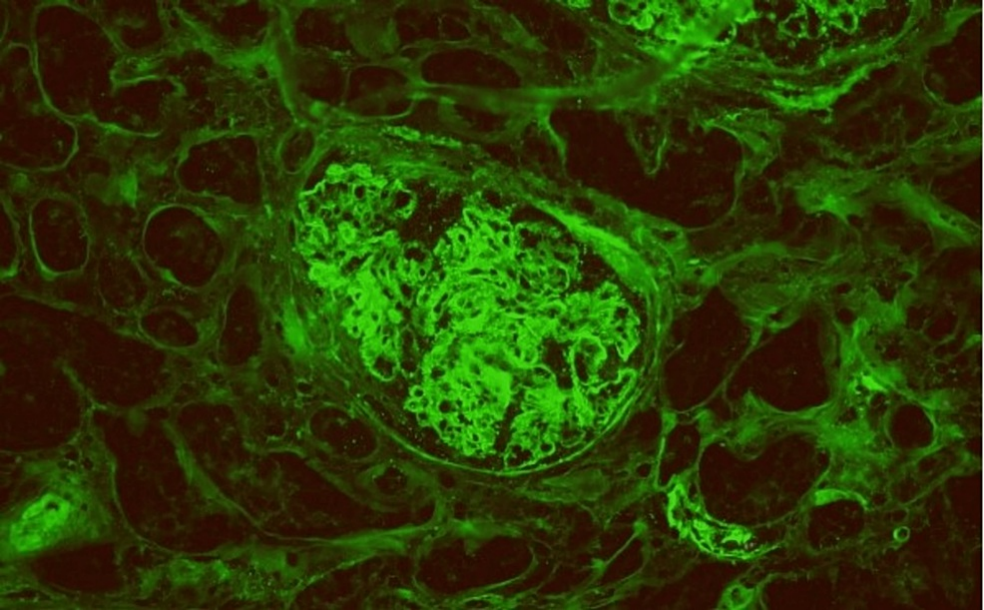
Immunofluorescence stain for IgG

**Figure 3:**
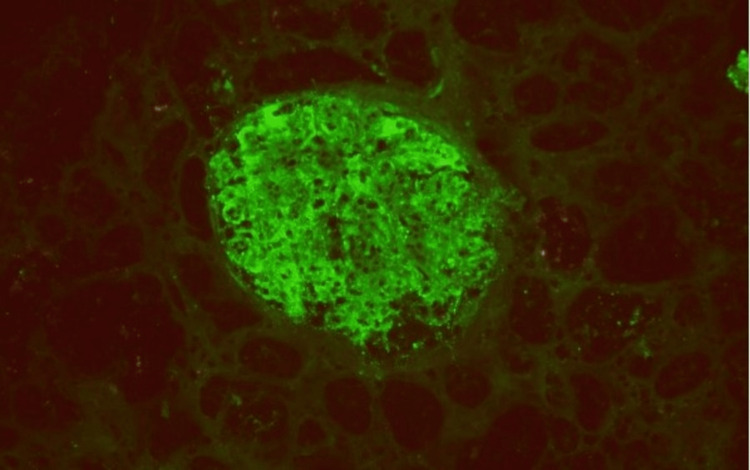
Immunofluorescence stains for C1q

**Figure 4:**
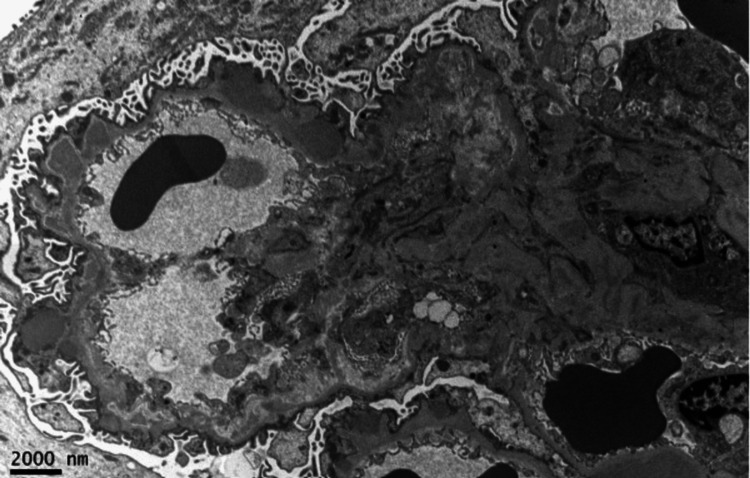
Electron micrograph demonstrating abundant electron-dense deposits in the mesangial and in the glomerular capillary wall including subepithelial, intramembranous, and subendothelial Several deposits have a moth-eaten appearance

Immunofluorescence revealed IgG (3+) (Figure 2), IgA (1+), IgM (1+), kappa (3+), lambda (2+), C3 (3+), and C1q (3+) (Figure 3) with coarse granular staining along the capillary wall and in the mesangial areas. Electron microscopy (EM) showed abundant electron-dense deposits in the mesangial as well as subepithelial deposits, some of which were large and hump-like. Intramembranous and subendothelial deposits were also seen (Figure 4).

These findings were consistent with immune complex glomerulonephritis with features of membranoproliferative glomerulonephritis (MPGN) with significant chronicity. This patient met the updated ACR/EULAR criteria for SLE, which requires fulfilling at least 10 points; this patient’s score was 21 and hence was very indicative of SLE. The patient started immunosuppression with mycophenolate mofetil and prednisone taper. The patient's condition improved as the proteinuria dropped to 500 mg/g, the creatinine returned to baseline, and the complements C3 and C4 returned to normal.

## Discussion

Glomerular full-house staining on immunofluorescence is a well-known characteristic feature of lupus nephritis [6]. Full-house pattern by immunofluorescence is defined as concurrent positive staining for IgA, IgG, IgM, C3, and C1q [7], though it is occasionally observed in other glomerular disorders as well. The earlier ACR criteria stated that diagnosing SLE required fulfilling at least four clinical and serological criteria [8]. Seronegative lupus nephritis was the term previously used to describe patients with kidney biopsy typical of lupus nephritis but without any other clinical or serological evidence of SLE [9,10].

In the revised ACR/EULAR criteria, ANA has been introduced as the entry criteria, and several items with weightage ranging from two to 10 have been included [11,12]. The importance of renal biopsy in individuals with suspected lupus nephritis is demonstrated by the fact that class III/IV lupus nephritis on biopsy has the highest weight at 10 points. Therefore, it is now possible to make a diagnosis based on a kidney biopsy thanks to the updated ACR/EULAR criteria. However, one should make sure that no alternative diagnosis is conceivable and rule out all other possibilities [11,13].

So far, 180 different autoantibodies have been detected in patients with SLE, thereby making it the autoimmune disease with the largest variety of detectable autoantibodies [14-17]. ANCA antibody prevalence varies in SLE. The prevalence was reported to be 37% [18] in a study by Pradhan et al., with a P-ANCA predominance as reported in our case. Anti-U1 RNP antibodies may be present in other autoimmune disorders with lesser titers, as was the case with our patient, but high titers are indicative of mixed connective tissue disease (MCTD) [19]. Patients with SLE can have anti-U1 RNP antibodies in up to 25-47% of cases [20].

## Conclusions

While the current diagnostic criteria for different autoimmune illnesses may serve to distinguish them as distinct disease entities to some extent, there is significant overlap frequently, underscoring our lack of understanding regarding these disease processes. The wide variety of clinical and immunological features in patients with Sjögren's syndrome often overlap with other autoimmune diseases, posing a diagnostic challenge. With the updated ACR/EULAR criteria, we expect that more SLE patients with characteristic findings of lupus nephritis on biopsy will receive a prompt diagnosis and undergo timely intervention.
